# Anthropometric Characteristics and Sex Influence Magnitude of Skin Cooling following Exposure to Whole Body Cryotherapy

**DOI:** 10.1155/2014/628724

**Published:** 2014-06-29

**Authors:** L. E. Hammond, S. Cuttell, P. Nunley, J. Meyler

**Affiliations:** ^1^Department of Sports Therapy and Rehabilitation, University of Bedfordshire, Park Square, Luton, Bedfordshire LU1 3JU, UK; ^2^Sports Studies, Moulton College, West Street, Moulton, Northampton NN3 7RR, UK

## Abstract

This study explored whether anthropometric measures influence magnitude of skin cooling following exposure to whole body cryotherapy (WBC). Height, weight, body fat percentage, and lean mass were measured in 18 male and 14 female participants. Body surface area, body surface area to mass ratio, body mass index, fat-free mass index, and fat mass index were calculated. Thermal images were captured before and after WBC (−60°C for 30 seconds, −110°C for 2 minutes). Skin temperature was measured at the chest, arm, thigh, and calf. Mean skin temperature before and after WBC and change in mean skin temperature (Δ*T*_sk_) were calculated. Δ*T*_sk_ was significantly greater in females (12.07 ± 1.55°C) than males (10.12 ± 1.86°C; *t*(30) = −3.09, *P* = .004). A significant relationship was observed between body fat percentage and Δ*T*_sk_ in the combined dataset (*P* = .002, *r* = .516) and between fat-free mass index and Δ*T*_sk_ in males (*P* = .005, *r* = .622). No other significant associations were found. Skin response of individuals to WBC appears to depend upon anthropometric variables and sex, with individuals with a higher adiposity cooling more than thinner individuals. Effects of sex and anthompometrics should be considered when designing WBC research or treatment protocols.

## 1. Introduction

Whole body cryotherapy (WBC) involves a short exposure to very cold air whilst wearing minimal clothing. It has been used in both clinical and sporting populations for treatment of depression [1], rheumatic conditions [2], ankylosing spondylitis [3], and exercise-induced muscle damage [4]. The thermodynamics of WBC centre on the principle of unidirectional heat transference from high to low temperatures [5], where body tissues lose their heat to the cryotherapy modality. The interaction between body and cryochamber occurs principally at the skin, and skin temperature directly reflects the balance of heat loss to the environment and heat deposited by metabolically active tissue [6]. Skin temperature has been shown to reduce significantly immediately after WBC exposure and then increase up to 1 hour after exposure, with concomitant decreases in muscle and core temperature [7, 8]. Heat transfer mechanisms and processes have not been previously examined with WBC, but, with application of ice packs, the transfer of heat from the body depends on several factors including size of contact area, the difference in starting temperatures, and the heat capacity of each material [9]. Thickness of adipose tissue also affects cooling time, with thicker skinfolds requiring longer ice exposure than thinner skin folds to produce a standard temperature in deeper tissues [10]. With WBC, differences in magnitude of cooling have been observed in individuals with a high body mass index (BMI) compared to low body mass index [6]. Collectively, the evidence from ice pack and WBC research suggests that standardised WBC protocols may result in differences in cooling in individuals with different anthropometric characteristics. To the best of our knowledge, no study has explored whether a range of anthropometric measures influence the magnitude of skin cooling when using WBC. Potentially important anthropometric factors include the size of contact area between cold air and skin (body surface area (BSA)), level of subcutaneous fat, or calculated ratios of anthropometry, for example, BMI or BSA to mass ratio (BSA : mass). Establishing this knowledge has implications for determining whether individualised doses based on anthropometric measures are warranted for WBC, as is found in other areas of medicine including the use of BSA for dosing chemotherapy [11].

Therefore the aims of this exploratory study were to

- examine the relationship between anthropometric measures and magnitude of skin cooling following exposure to WBC;
- explore whether anthropometric measures can be used to predict magnitude of skin cooling following exposure to WBC;
- compare skin cooling responses to WBC in males and females.

## 2. Materials and Methods

### 2.1. Participants

Thirty-two recreationally active participants (males: *n* = 18, mean age: 29.5 ± 4.4 years; females *n* = 14, mean age: 28.3 ± 6.4 years) gave written informed consent to participate in the study. Each participant completed a medical form and declared that they were free from medical conditions including Raynaud's phenomenon and other cold sensitivities, heart conditions, claustrophobia, and allergy to adhesive tape. The study was approved by the Moulton College Research Ethics Committee in accordance with the Declaration of Helsinki.

### 2.2. Instrumentation and Measures

Height and weight were measured using laboratory scales and stadiometer. Body composition was measured using bioelectrical impedance (BIA) at a frequency of 50 kHz (Biostat 1500, Isle of Man) yielding information on body fat percentage and lean mass. From the anthropometric measures taken, height and weight, BMI, body surface area using the Du Bois equation [12], and BSA : mass were also calculated. As the BMI concept has been criticised for not accounting effectively for body composition, fat-free mass index (FFMI; fat-free mass/height^2^) and fat mass index (FMI; fat mass/height^2^) were also calculated [13]. A summary of the anthropometric characteristics of the participants can be seen in Table 1.

Skin temperature was measured by taking thermal images using a factory calibrated FLIR Thermal Imaging Camera (E40BX FLIR systems, Danderyd, Sweden). The thermographs were taken according to the standard protocol for infrared imaging in medicine [14]. Thermal imaging has been shown to be reproducible between users, although being variable within individuals over time [15], and was suggested to be an accurate and reliable method for assessing skin temperature following cryotherapy in a recently published review [16]. The camera was positioned on a tripod, 3 metres from the participants, with an emissivity factor of 0.98 which is appropriate for skin [16, 17]. The thermographs were later analysed using FLIR QuickReport to establish mean skin temperature within regions of interest (ROI) on the chest, anterior thigh, posterior upper arm, and calf, for both pre-WBC (pre*T*_sk_) and post-WBC (post*T*_sk_) images. Mean skin temperature (*T*_sk_) was calculated from four body sites using the equation of Ramanathan [18]. The change in skin temperature (Δ*T*_sk_) from before WBC to after WBC (outcome variable) was calculated by subtracting post*T*_sk_ from pre*T*_sk_.

Cryotherapy exposures took place in a liquid nitrogen cooled cryogenic chamber at The Chris Moody Sports Injury and Rehabilitation Centre in Northamptonshire, United Kingdom. The unit was purpose-built and temperature controlled (Juka, Poland), comprising two chambers (−60°C and −110°C) connected by an internal door.

### 2.3. Procedure

Participants were instructed to abstain from consuming caffeine, smoking, or taking part in exercise on the day of testing and had not consumed food within 2 hours of WBC exposure. They were asked to remain hydrated. Height and weight were first measured; then participants acclimated for 20 minutes to ambient room temperature. Four-electrode BIA was undertaken and connected at two sites (wrist/hand and ankle/foot).

In preparation to enter the cryotherapy chambers, participants undressed to shorts (males) and shorts and vest (females). Protective garments worn by the participants included gloves, socks, clogs, tubular bandages to cover elbows and knees, headband to cover the ears, and surgical mask over the mouth. Glasses, jewellery, and piercings were removed before entering the chamber. Thermally inert markers were attached using adhesive tape to eight sites on the participants' bodies to create ROI for temperature analysis, in a similar fashion to that described by Costello et al. [16]. The sites used were the acromion process, olecranon process, coracoid process, 5 cm below the sternoclavicular joint, anterior superior iliac spine, 5 cm above the patella, popliteal crease, and the distal musculotendinous junction of gastrocnemius. Participants were instructed to stand in anatomical position in a thermally controlled room (21.6 ± 1.6°C), and two pre-WBC thermographic images of the whole body (anterior and posterior) were taken.

Following a safety briefing from the cryochamber operators, participants (in pairs) entered the antechamber for 30 seconds at −60 ± 4.7°C and transferred through an internal door to the main chamber for 2 minutes at −110 ± 2.3°C. This replicates commonly reported time and temperature protocols [2, 19–23]. At the completion of the WBC exposure, participants transferred immediately to the adjacent thermally controlled area to capture post-WBC thermal images.

### 2.4. Statistical Analysis

Descriptive analysis of pre- and post-WBC temperatures for each of the ROI and *T*_sk_ measures was undertaken. Differences in mean decrease in *T*_sk_ between sexes (Δ*T*_sk_) were examined using an independent *t*-test, with significance accepted at *P* ≤ .05. Bivariate correlation between six anthropometric measures (BMI, FFMI, FMI, BSA, body fat percentage, and BSA : mass) and Δ*T*_sk_ were calculated using a Pearson's correlation coefficient (*r*). A Bonferroni adjusted alpha value of 0.008 was used as multiple correlations were performed [24]. For significantly associated variables, simple linear regression was calculated.

## 3. Results 

### 3.1. Analysis of Change in Δ*T*_sk_ after WBC

The mean temperatures of all body regions (*T*_Chest_, *T*_Arm_, *T*_Thigh_, and *T*_Calf_) and of *T*_sk_ decreased after exposure to whole body cryotherapy in both males and females (Table 2). The lowest temperatures reached were in the calf (19.54 ± 3.28 and 15.05 ± 2.04°C for males and females, resp.). Δ*T*_sk_ was significantly greater in females (12.07 ± 1.55°C) than males (10.12 ± 1.86°C), *t*(30) = −3.09, *P* = .004. When considering the data set as a whole, Δ*T*_sk_ was 11.0 ± 1.96°C, equivalent to a 34.2% decrease in skin temperature from pre- to post-WBC exposure.

### 3.2. Exploring Relationships between Anthropometric Measures and Δ*T*_sk_

**Figure 1:**
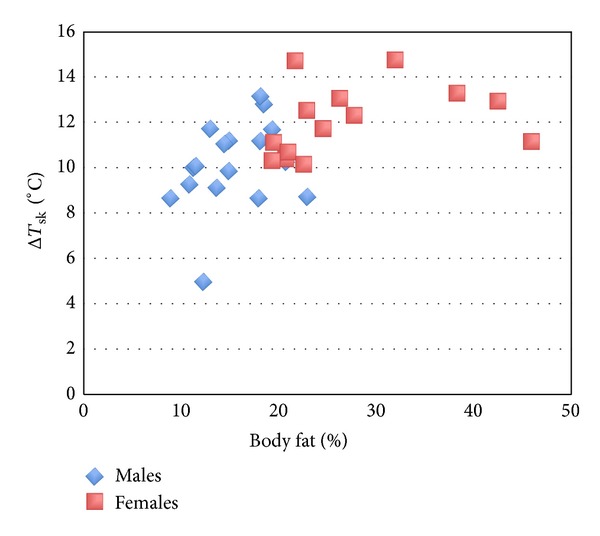
Significant association between body fat percentage and Δ*T*_sk_ in combined males and females.

**Figure 2:**
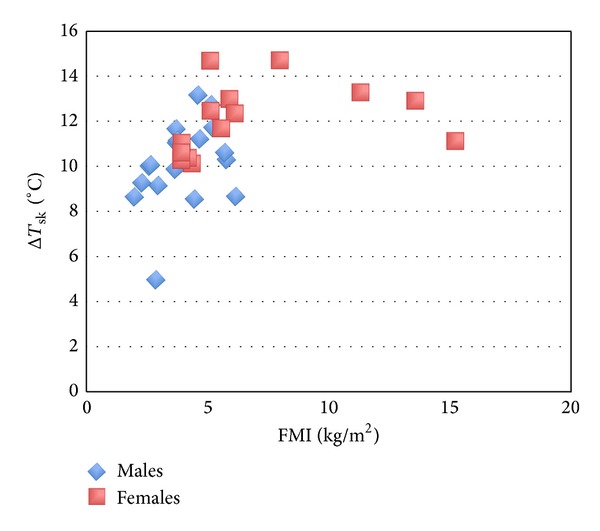
Nonsignificant association between FMI and Δ*T*_sk_ which demonstrates a highly similar pattern to that observed with body fat percentage and Δ*T*_sk_.

**Figure 3:**
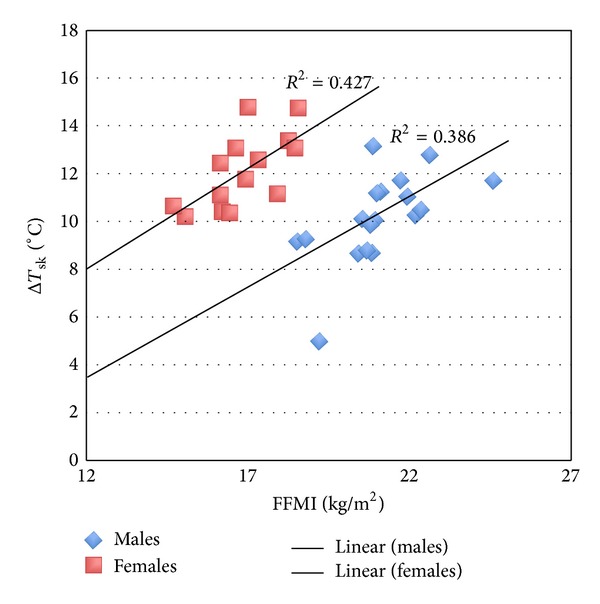
Association between FFMI and Δ*T*_sk_ which was significant for males but not females.

For combined male and female data, a highly significant moderate relationship was observed between body fat percentage and Δ*T*_sk_ (*P* = .002, *r* = .516).  Figure 1 shows the effect of adiposity on Δ*T*_sk_, with females demonstrating higher levels of both adiposity and cooling than males. A very similar relationship is seen between FMI and Δ*T*_sk_ (Figure 2), although this is not significant. No other anthropometric variables were significantly associated in the combined group (Table 3). A significant moderate association was observed for FFMI with Δ*T*_sk_ in males (*P* = .005, *r* = .622) and an effect of sex and FFMI appears to be present (Figure 3), where the two sexes are distinctly clustered along similarly sloped lines with different intercepts. Δ*T*_sk_ increased with FFMI in both male and female participants, but the female cluster of data points are situated to the left on the graph, indicating that, for a given FFMI, females cooled more than males. There were no significant associations between Δ*T*_sk_ and anthropometric measures in females.

### 3.3. Examining Predictors for Δ*T*_sk_

Simple linear regression of the significantly associated variable of body fat percentage with Δ*T*_sk_ gives the following regression equation to predict magnitude of cooling in males and females:

(1) $$\begin{array}{c} \Delta T_{\text{sk}}=0.12\;\left(\text{body}\;\text{fat}\;\text{percentage}\right)+8.6\;\left(\pm 1.7\right). \end{array}$$

Similarly, FFMI can be used as a predictor of Δ*T*_sk_ in males using the following equation:

(2) $$\begin{array}{c} \Delta T_{\text{sk}}=0.81\;\left(\text{FFMI}\right)-6.9. \end{array}$$

## 4. Discussion 

The response of individuals to WBC appears to depend upon sex and anthropometric variables, with body fat percentage demonstrating a significant positive correlation with Δ*T*_sk_ in a combined dataset of males and females. Females had higher levels of adiposity than males and experienced a greater degree of skin cooling. A significant difference was observed for Δ*T*_sk_ in males and females, with females' skin cooling more than that of males, suggesting that optimal WBC dosage may differ between sexes. Differences in local skin cooling were also observed, with the greatest difference found in the calf, where Δ*T* was almost 4°C more in females. The differences observed between males and females might be explained by anthropometric and thermoregulatory differences; females have 20% smaller body mass, 14% more fat, 33% less lean body mass and 18% less surface area than males [25], a higher subcutaneous to visceral fat ratio [26], and a smaller ratio of FMI to FFMI than males [13]. Furthermore, females' BSA : mass is higher than males, and the greater the ratio of BSA to mass is the greater the heat lost is [27]. Under cold stress, females have a more extensively vasoconstricted periphery, with greater surface heat losses [25] and show a significantly reduced sensitivity of the shivering response [28].

FFMI was significantly associated with Δ*T*_sk_ for male participants and appeared to also demonstrate a trend in females although this was not significant. Similarly, BMI (in males) and FMI (in males and females combined) appeared to demonstrate trends that were not significant; however the lack of statistical significance may be explained by the very conservative Bonferroni adjusted alpha value used in this study, potentially increasing the risk of Type II error. These factors suggest that adiposity and sex alone do not fully explain the differences in cooling observed; rather there appears to be an effect where the bigger the individual is (be that through body fat percentage, BMI, or either of its two constituents, FMI or FFMI), the greater the amount of heat they lose at the skin following WBC. Research examining the effects of extreme cold on the skin in individuals of different levels of fatness is scant, as researchers have mainly focused on core temperature and tolerance to cold environments. However, some explanations have been offered that could explain the findings reported here. Matsumoto et al. [29] reported that obese women displayed significantly lower sympathetic responsiveness to cold air (10°C) than nonobese women, which would result in a greater degree of cutaneous heat loss following the reduced degree of vasoconstriction. Alternatively, individuals with higher subcutaneous fat stores tend to have a lower *T*_sk_ than their thinner counterparts as fat insulates the skin from the warming effect of deeper tissues [27]. A similar relationship between BMI and Δ*T*_sk_ following WBC has previously been reported in a sample of males and females [6], where individuals with a higher BMI cooled more than those with a lower BMI. Despite some differences in study protocols and subject characteristics, those findings corroborate the findings presented here.

Skin temperatures recorded at the different anatomical sites sampled for combined sexes showed a trend that is also in agreement with previous work in this area. Δ*T* was greater in the lower extremities (calf 14.3°C; thigh 12.2°C) than the chest or forearm (8.5°C and 10.5°C, resp.), which has also been shown in previous cryotherapy studies [6, 30].

### 4.1. Methodological Considerations

Like many studies of WBC, skin temperature was measured by infrared thermography in this study. Infrared cameras have the advantage over other methods of assessing skin temperature (such as thermocouples, thermistors, or wireless sensors) that they do not require contact with the skin and therefore do not create any local area of insulation [16]. However, no verification of the temperatures recorded was made during this study, and due to the lack of agreed guidelines on the creation of ROI the regions used for this study may not be directly comparable with those used in other studies.

BIA is a popular commercial method for measuring body composition and has been shown to be reliable and valid when compared to skinfold analysis and hydrostatic weighing [31]. However it can be affected by several factors including hydration, food consumption, and exercise. Participants in the study were required to remain hydrated, to have abstained from eating within the two hours prior to WBC exposure, and to have abstained from any exercise that day in order to minimise these effects. Despite this, it is possible that the limitation of using BIA compared to a gold standard or reference measure of body composition may have adversely affected these data. Hydration status could also theoretically influence heat dissipation after WBC, and future work could address this additional factor.

Skin temperature is clearly an important parameter in influencing internal cooling following WBC [7]; however it is a limitation of this study that it is unknown whether there were differences present in core temperature as a result of the differences in *T*_sk_ observed between individuals. Core temperature has previously been shown to display a predictable relationship with *T*_sk_ following WBC [8] and this would be an interesting area for future study in order to further explain the findings reported here. Further research using a measure of core temperature such as a rectal probe or temperature-sensitive ingestible capsule would be needed to make those comparisons, which were not available for this study. Furthermore, time-course data would be required to evaluate if rewarming of the skin and core varied between individuals of different anthropometry.

One issue that was not accounted for in female subjects was potential variation in basal body temperature according to the phase of the menstrual cycle that individuals were in at the time of testing. Some attempt was made by the research team to quantify the cycle phase to explore whether it was confounding; however three-quarters of the female participants were using hormonal contraception, and consequently some participants had not experienced menses for some time. Therefore it was not possible to examine any potential effects of this variable. The effects of this on the findings are likely to be minimal and, however, should be taken into consideration when interpreting the female portion of the data presented here.

The sample size used for this study is greater than many studies that have explored the effects of WBC; however at 32 it was still relatively small when considering a heterogeneous group of people. The female dataset was more heterogeneous than the male dataset, with large standard deviations observed for mass, BMI, and body fat percentage. A larger number of participants would result in a more representative study population, and further research with much larger numbers of participants is needed to validate the findings presented here. A larger sample size would also facilitate performing multiple regressions on the data to explore whether multiple anthropometric predictors exist for skin cooling following WBC; this was not possible in this data set as the test would be underpowered for 6 predictors in 32 participants. Further research should also examine whether the same effects occur in athletic populations, where anthropometric characteristics are diverse and where WBC has become a popular tool for recovery [3].

### 4.2. Practical Implications and Conclusions

The findings of this study appear to suggest that individuals with different anthropometric characteristics receive different therapeutic effects in terms of changes in skin temperature from identical doses of WBC, with individuals with higher adiposity cooling more at the skin than thinner individuals. Similarly, males and females appear to respond differently to WBC exposure, and this should be considered when designing and interpreting research studies and determining commercial treatment protocols for WBC. These findings could have implications for the individualisation of dosage for WBC. Personalised medicine is becoming an increasingly popular model of healthcare and these findings lend support to the idea of individualising dosage, although more research would be needed to determine a multitude of parameters in order to achieve this. As well as application for medical usage, this may have important implications in determining WBC dosage in elite athletic populations, for those that have access to cryotherapy chambers. Performance margins are very small in elite sport, so individualising a dose of WBC may assist in creating optimal effects in individual elite athletes. Manipulating the parameters of time and temperature of exposure adjusts the therapeutic dose given to users, but the question of “what are the optimal modality, temperature, and duration required to elicit the required physiological response?” posed by Costello et al. [7] highlights the current “holy grail” in WBC research. As presently the optimal WBC dose (in terms of duration and temperature) has yet to be elucidated, manipulation of dose based on individual characteristics to achieve optimal effects might be a goal for the future. However, further work should be conducted towards this to help better understand the use and application of WBC in different populations.

## Figures and Tables

**Table 1:** Anthropometric characteristics of study participants.

	Males		Females	
	Mean	SD	Mean	SD
Height (cm)	179.2	6.2	164.6	6.5
Mass (kg)	80.6	9.4	64.1	12.4
BMI (kg/m^2^)	25.0	2.3	23.7	4.6
FFMI (kg/m^2^)	21.1	1.4	16.8	1.2
FMI (kg/m^2^)	4.0	1.3	6.9	3.8
BSA (m^2^)	2.0	0.1	1.7	0.2
Lean mass (kg)	67.7	6.2	45.7	4.9
Body fat percentage (%)	15.6	4.0	27.4	8.8
BSA : Mass (cm^2^/kg)	2.0	0.1	3.0	0.3

**Table 2:** Mean temperature of body regions (°C) before and after exposure to WBC.

	Males			Females			Combined		
	Before WBC (°C)	After WBC (°C)	Δ*T* (°C)	Before WBC (°C)	After WBC (°C)	Δ*T* (°C)	Before WBC (°C)	After WBC (°C)	Δ*T* (°C)
*T*_Chest_	33.58 ± 0.76	25.45 ± 1.97	8.13 ± 1.57	34.15 ± 0.73	25.25 ± 2.98	8.90 ± 2.48	33.83 ± 0.79	25.3 ± 2.42	8.47 ± 2.02
*T*_Arm_	31.17 ± 1.14	21.20 ± 2.00	9.98 ± 1.81	31.52 ± 1.07	20.40 ± 2.40	11.13 ± 1.84	31.33 ± 1.10	20.85 ± 2.22	10.48 ± 1.88
*T*_Thigh_	31.47 ± 1.14	20.50 ± 2.56	10.97 ± 2.36	31.23 ± 0.86	17.38 ± 1.76	13.85 ± 1.21	31.36 ± 1.02	19.14 ± 2.72	12.23 ± 2.41
*T*_Calf_	32.20 ± 0.57	19.54 ± 3.28	12.66 ± 3.06	31.50 ± 0.76	15.05 ± 2.04	16.46 ± 1.96	31.9 ± 0.74	17.57 ± 3.57	14.33 ± 3.2
*T*_sk_	***32.16 ± 0.77***	***22.0 ± 2.10***	***10.12 ± 1.86***	***32.25 ± 0.73***	***20.18 ± 2.11***	***12.07 ± 1.55***	***32.2 ± 0.74***	***21.20 ± 2.26***	***11.0 ± 1.96***

**Table 3:** *r* values and significance of correlations of anthropometric measures with Δ*T*_sk_.

	Males		Females		Combined	
	*r*	*P*	*r*	*P*	*r*	*P*
BMI	.590	.010	.402	.154	.286	.113
FFMI	.622	.005∗	.653	.009	−.013	.478
FMI	.380	.118	.288	.315	.444	.010
BSA	.115	.649	.417	.138	−.216	.234
Body fat %	.326	.187	.313	.276	.516	.002∗
BSA : Mass	−.512	.030	−.501	.068	−.137	.455

*Statistically significant (≤.008).

## References

[1] Rymaszewska J, Tulczynski A, Zagrobelny Z, Kiejna A, Hadrys T (2003). Influence of whole body cryotherapy on depressive symptoms—preliminary report. *Acta Neuropsychiatrica*.

[2] Hirvonen HE, Mikkelson MK, Kautiainen H, Pohjolainen TH, Leirsalo-Repo M (2006). Effectiveness of different cryotherapies on pain and disease activity in active rheumatoid arthritis. A randomised single blinded controlled trial. *Clinical and Experimental Rheumatology*.

[3] Banfi G, Lombardi G, Colombini A, Melegati G (2010). Whole-body cryotherapy in athletes. *Sports Medicine*.

[4] Hausswirth C, Louis J, Bieuzen F (2011). Effects of whole-body cryotherapy vs. far-infrared vs. passive modalities on recovery from exercise-induced muscle damage in highly-trained runners. *PLoS ONE*.

[5] Westerlund T, Oksa J, Smolander J, Mikkelsson M (2003). Thermal responses during and after whole-body cryotherapy (−110°C). *Journal of Thermal Biology*.

[6] Cholewka A, Stanek A, Sieroń A, Drzazga Z (2012). Thermography study of skin response due to whole-body cryotherapy. *Skin Research and Technology*.

[7] Costello JT, Culligan K, Selfe J, Donnelly AE (2012). Muscle, skin and core temperature after −110°C cold air and 8°C water treatment. *PloS ONE*.

[8] Selfe J, Alexander J, Costello JT (2014). The effect of three different (−135°C) whole body cryotherapy exposure durations on elite rugby league players. *PLoS ONE*.

[9] Merrick MA, Jutte LS, Smith ME (2003). Cold modalities with different thermodynamic properties produce different surface and intramuscular temperatures. *Journal of Athletic Training*.

[10] Otte JW, Merrick MA, Ingersoll CD, Cordova ML (2002). Subcutaneous adipose tissue thickness alters cooling time during cryotherapy. *Archives of Physical Medicine and Rehabilitation*.

[11] Gurney H (2002). How to calculate the dose of chemotherapy. *British Journal of Cancer*.

[12] Du Bois D, Du Bois EF (1916). A formula to estimate the approximate surface area if height and weight be known. *Archives of Internal Medicine*.

[13] Schutz Y, Kyle UUG, Pichard C (2002). Fat-free mass index and fat mass index percentiles in caucasians aged 18–98 y. *International Journal of Obesity*.

[14] Ring EF, Ammer K (2000). The technique of infrared imaging in medicine. *Thermology International*.

[15] Zaproudina N, Varmavuo V, Airaksinen O, Närhi M (2008). Reproducibility of infrared thermography measurements in healthy individuals. *Physiological Measurement*.

[16] Costello JT, McInerney CD, Bleakley CM, Selfe J, Donnelly AE (2012). The use of thermal imaging in assessing skin temperature following cryotherapy: a review. *Journal of Thermal Biology*.

[17] Steketee J (1973). Spectral emissivity of skin and pericardium. *Physics in Medicine and Biology*.

[18] Ramanathan NL (1964). A new weighting system for mean surface temperature of the human body. *Journal of Applied Physiology*.

[19] Klimek AT, Lubkowska A, Szyguła Z, Fra̧czek B, Chudecka M (2011). The influence of single whole body cryostimulation treatment on the dynamics and the level of maximal anaerobic power. *International Journal of Occupational Medicine and Environmental Health*.

[20] Gong W, Ma S, Ro H (2011). Effect of whole body cryotherapy with spinal decompression on cervical disc herniation by digital infrared thermal imaging. *Journal of Physical Therapy Science*.

[21] Leppäluoto J, Westerlund T, Huttunen P (2008). Effects of long-term whole-body cold exposures on plasma concentrations of ACTH, beta-endorphin, cortisol, catecholamines and cytokines in healthy females. *Scandinavian Journal of Clinical and Laboratory Investigation*.

[22] Smolander J, Westerlund T, Uusitalo A, Dugué B, Oksa J, Mikkelsson M (2006). Lung function after acute and repeated exposures to extr emely cold air (−110°C) during whole-body cryotherapy. *Clinical Physiology and Functional Imaging*.

[23] Dugué B, Smolander J, Westerlund T (2005). Acute and long-term effects of winter swimming and whole-body cryotherapy on plasma antioxidative capacity in healthy women. *Scandinavian Journal of Clinical and Laboratory Investigation*.

[24] Curtin F, Schulz P (1998). Multiple correlations and Bonferroni's correction. *Biological Psychiatry*.

[25] Burse RL (1979). Sex differences in human thermoregulatory response to heat and cold stress. *Human Factors*.

[26] Enzi G, Gasparo M, Raimondo Biondetti P, Fiore D, Semisa M, Zurlo F (1986). Subcutaneous and visceral fat distribution according to sex, age, and overweight, evaluated by computed tomography. *American Journal of Clinical Nutrition*.

[27] Stocks JM, Taylor NAS, Tipton MJ, Greenleaf JE (2004). Human physiological responses to cold exposure. *Aviation Space and Environmental Medicine*.

[28] Grucza R, Pekkarinen H, Hänninen O (1999). Different thermal sensitivity to exercise and cold in men and women. *Journal of Thermal Biology*.

[29] Matsumoto T, Miyawaki T, Ue H, Kanda T, Zenji C, Moritani T (1999). Autonomic responsiveness to acute cold exposure in obese and non-obese young women. *International Journal of Obesity*.

[30] Herrera E, Sandoval MC, Camargo DM, Salvini TF (2010). Motor and sensory nerve conduction are affected differently by ice pack, ice massage, and cold water immersion. *Physical Therapy*.

[31] Jackson AS, Pollock ML, Graves JE, Mahar MT (1988). Reliability and validity of bioelectrical impedance in determining body composition. *Journal of Applied Physiology*.

